# Suppression of Cell-Mediated Immunity following Recognition of Phagosome-Confined Bacteria

**DOI:** 10.1371/journal.ppat.1000568

**Published:** 2009-09-04

**Authors:** Keith S. Bahjat, Nicole Meyer-Morse, Edward E. Lemmens, Jessica A. Shugart, Thomas W. Dubensky, Dirk G. Brockstedt, Daniel A. Portnoy

**Affiliations:** 1 Earle A. Chiles Research Institute, Robert W. Franz Cancer Research Center, Providence Cancer Center, Portland, Oregon, United States of America; 2 Department of Molecular and Cell Biology, University of California, Berkeley, California, United States of America; 3 Anza Therapeutics, Concord, California, United States of America; 4 School of Public Health, University of California, Berkeley, California, United States of America; University of Toronto, Canada

## Abstract

*Listeria monocytogenes* is a facultative intracellular pathogen capable of inducing a robust cell-mediated immune response to sub-lethal infection. The capacity of *L. monocytogenes* to escape from the phagosome and enter the host cell cytosol is paramount for the induction of long-lived CD8 T cell–mediated protective immunity. Here, we show that the impaired T cell response to *L. monocytogenes* confined within a phagosome is not merely a consequence of inefficient antigen presentation, but is the result of direct suppression of the adaptive response. This suppression limited not only the adaptive response to vacuole-confined *L. monocytogenes*, but negated the response to bacteria within the cytosol. Co-infection with phagosome-confined and cytosolic *L. monocytogenes* prevented the generation of acquired immunity and limited expansion of antigen-specific T cells relative to the cytosolic *L. monocytogenes* strain alone. Bacteria confined to a phagosome suppressed the production of pro-inflammatory cytokines and led to the rapid MyD88-dependent production of IL-10. Blockade of the IL-10 receptor or the absence of MyD88 during primary infection restored protective immunity. Our studies demonstrate that the presence of microbes within a phagosome can directly impact the innate and adaptive immune response by antagonizing the signaling pathways necessary for inflammation and the generation of protective CD8 T cells.

## Author Summary

Little is understood about how the immune system distinguishes between pathogenic and non-pathogenic microbes. Limiting or preventing infections by intracellular pathogens requires the activation of innate immunity and the consequent generation of effector and memory T cells, which recognize and kill infected cells. Investigators are currently testing attenuated versions of pathogenic microbes as vaccines in an attempt to generate pathogen-specific T cells without causing disease. Unfortunately, attenuated microbes often fail to elicit long-lived protective immunity. We hypothesized that attenuated bacterial vaccines do not immunize because they fail to activate a stimulatory arm of host innate immune receptors. However, we found that these attenuated bacterial vaccines are not simply prevented from activating immunity, but rather generate a negative signal that inhibits the desired immune response. These studies may explain why the addition of an adjuvant to ineffective vaccines does not necessarily improve immunogenicity. Furthermore, these studies provide a framework for the development of attenuated vaccines that do not inhibit the desired immune responses.

## Introduction

The intracellular bacterium *L. monocytogenes* has been studied for decades as a model of innate and cellular immunity [1]. Infection with *L. monocytogenes* leads to a robust innate and adaptive response, characterized by the generation of long-lived antigen-specific CD4 and CD8 T cells [2], the latter of which are predominantly responsible for protective immunity [3],[4]. Following engulfment by the host cell, *L. monocytogenes* escapes from the phagosome and into the host cell cytosol via secretion of the pore-forming cytolysin, listeriolysin O (LLO) [5]. Once within the cytosol, the bacteria express ActA that facilitates cell to cell spread via polymerization of host-cell actin [6]. ActA-deficient mutants still induce protective immunity, while mutants lacking LLO (sometimes designated as Δ*hly*) elicit an antigen-specific T cell response, but these T cells are unable to provide protective immunity [7],[8]. Escape of *L. monocytogenes* into the cytosol permits bacterial growth and facilitates the MyD88-independent activation of a cytosolic surveillance pathway, leading to the production of a unique array of cytokines, including type I IFN [9]–[12]. What remains unclear is why *L. monocytogenes*, which contains ligands for multiple Toll-like receptors found on the cell surface and within the phagosome, only elicits effective adaptive immunity when entering the host cell cytosol [13],[14].

Innate immune recognition of *L. monocytogenes* is critical for controlling early microbial replication [2]. Interaction of the bacterium with host pattern recognition receptors (PRR) triggers a cascade of cytokines and chemokines that both recruits and arms innate immune effectors [15],[16]. *L. monocytogenes* contains ligands for TLR2 (peptidoglycan, lipotechoic acid and lipoproteins), TLR5 (flagellin), TLR9 (CpG motifs), and NOD2 (muramyl dipeptide), all of which may elicit proinflammatory cytokine secretion [17]–[22]. Rapid secretion of chemokines such as MCP-1 and MCP-3, and cytokines such as IFN-γ and TNF are essential for enhancing the recruitment and bacteriocidal functions of macrophages and neutrophils, which act to restrict bacterial burden prior to the onset of the adaptive response [23]–[25]. Typically suppressive cytokines such as IL-10 are also elicited in response to *L. monocytogenes* infection where they may contribute to bacterial persistence as well as T cell potency [26]–[28]. The innate response to these PRR-ligands also serves to shape the ensuing adaptive immune response [29]. Innate inflammatory cytokines produced in response to *L. monocytogenes* infection facilitate dendritic cell (DC) maturation and migration to the infection-associated secondary lymphatics [29],[30]. Maturation is essential for enhancing the stimulatory capacity of the DC via upregulation of costimulatory surface molecules and cytokines (e.g. CD80/86, CD70, IL-12p70, IL-18, IFN-α/β) [31]. Maturation also facilitates migration of the DC into the draining lymph node where it can interact with large numbers of naïve T cells [32]. Together, the local cytokine milieu and dendritic cell maturation state significantly contribute to the outcome of the DC-T cell interaction and ultimately, the potency of the T cell response [33].

We questioned how the response to a bacterium confined within a phagosome would impact the adaptive response to a bacterium within the host cell cytosol. Mice were infected with two distinct strains of *L. monocytogenes*. The first strain, ActA-Lm, escapes into the host cell cytosol and elicits long-lived CD8 T cell-dependent protective immunity [23],[34]. Because it cannot spread between cells, ActA-Lm is highly attenuated in vivo, can be administered at a higher dose, and is rapidly cleared from both liver and spleen (relative to wild-type *L. monocytogenes*). A second strain, LLO-Lm, is unable to produce listeriolysin O (LLO), and thus cannot escape from the phagosome [35]. Importantly, infection with LLO-Lm elicits CD8 T cells, but little or no protective immunity to a lethal wild-type *L.* monocytogenes challenge [7],[8]. To facilitate enumeration of *L. monocytogenes*-specific CD8 and CD4 T cell responses following infection, we used strains expressing chicken ovalbumin (OVA) fused to a non-lytic fragment of LLO.

## Results

### Phagosome-confined *L. monocytogenes* negatively impact protective immunity

**Figure 1:**
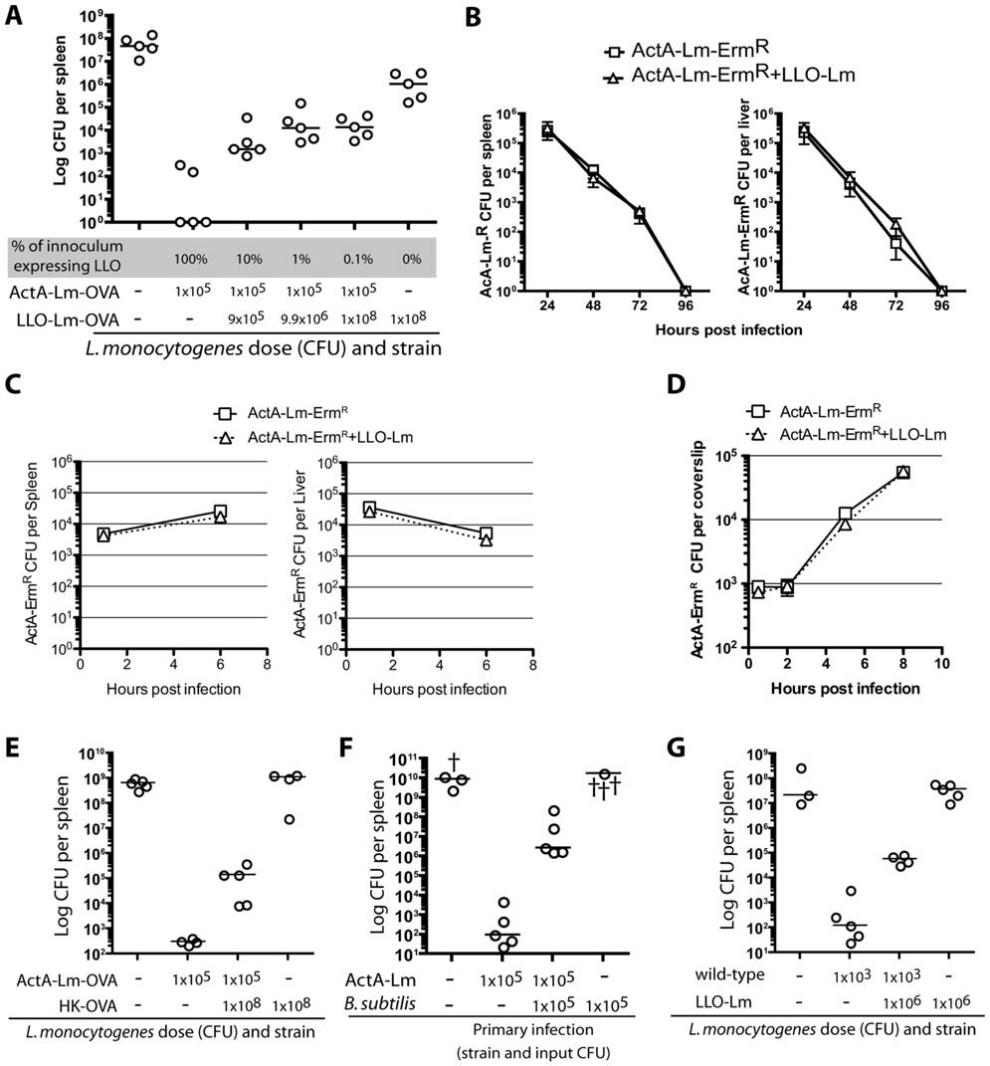
*L. monocytogenes* within a phagosome impairs protective immunity. Mice were infected with 1×10^5^ CFU ActA-Lm–OVA alone, or in combination with increasing doses of phagosome-confined LLO-Lm-OVA. (A) Mice were challenged 60 days later with a lethal dose of wt *L. monocytogenes*-OVA. Spleens were harvested 3 days later and CFU per spleen determined. (B) Mice were infected with 1×10^5^ CFU ActA-Lm-Erm^R^ alone, or in combination with 1×10^8^ CFU LLO-Lm. Erythromycin-resistant colonies were enumerated from the spleen and liver over 96 hours. Each data point represents the mean and standard error of 5 mice per group. (C) Mice were infected with 1×10^5^ CFU ActA-Lm-Erm^R^ alone, or in combination with 1×10^8^ CFU LLO-Lm. Erythromycin-resistant colonies were enumerated from the spleen and liver at 1 and 6 hours post infection. Each data point represents the mean and standard error of 5 mice per group from one representative experiment of two. (D) Bone marrow-derived macrophages were infected with ActA-Lm-Erm^R^ alone (at 1∶10,000), or in combination with 1×10^8^ CFU LLO-Lm (at 1∶200). Erythromycin-resistant colonies were enumerated at the indicated timepoints. Each data point represents the mean and standard error of 3 independent coverslips per timepoint from one representative experiment of two. (E) Mice were infected with the indicated combinations of 1×10^5^ CFU ActA-Lm–OVA and 1×10^8^ heat-killed ActA-Lm–OVA. 30 days later, mice were challenged with 1×10^5^ CFU of wt *L. monocytogenes*-OVA. Spleens were harvested 3 days later and CFU per spleen determined. (F) Mice were infected with 1×10^5^ CFU ActA-Lm, 1×10^5^ CFU *B. subtilis*, or the combination of both strains. 30 days post infection, mice were challenged and CFU determined. (G) Mice were infected with the indicated combinations of 1×10^3^ CFU wild-type and 1×10^6^ CFU LLO-Lm. 58 days later, mice were challenged with 1×10^5^ CFU of wild-type *L. monocytognes*. Spleens were harvested 3 days later and CFU per spleen determined. In all panels, each point represents a single animal with † indicating animals that died before CFU were determined. Lines indicate the median of each group.

To better understand the impact of phagosome-confined bacteria on the adaptive immune response we infected cohorts of mice with an identical dose of ActA-Lm-OVA (1×10^5^ colony forming units (CFU)), a dose sufficient to elicit long-lived CD8 T cell-mediated protective immunity. To this inoculum, we added increasing numbers of phagosome-confined LLO-Lm-OVA. We assessed protective immunity 60 days later by challenging with wild-type-*L. monocytogenes*-OVA, and then enumerating CFU in the spleen (Figure 1A). Strikingly, the protective immunity typically elicited by ActA-Lm-OVA was compromised by the presence of phagosome-confined LLO-Lm-OVA during primary infection. In other words, despite a significant increase in antigen during primary infection, the adaptive response to the cytosolic bacterium was impaired by the presence of bacteria within a phagosome. A potential explanation for this finding was that the addition of LLO-Lm-OVA to the inoculum facilitated more rapid clearance of ActA-Lm-OVA, decreasing the duration of antigen presentation and negatively impacting T cell potency. To test this hypothesis, we used an erythromycin-resistant strain of ActA-Lm (ActA-Lm-Erm^R^) combined with a large number of LLO-Lm (1×10^8^ CFU). We followed the clearance of the ActA-Lm-Erm^R^ strain by enumerating CFU on agar containing erythromycin (Figure 1B, C and D). Importantly, the addition of LLO-Lm did not impact the rate at which ActA-Lm-Erm^R^ were cleared from the spleen or liver or the in vitro growth rate within bone marrow-derived macrophages.

To determine whether the phagosome-confined bacteria required metabolic activity, heat-killed *L. monocytogenes* were added to an inoculum of ActA-Lm (Figure 1E). Similar to our observations with LLO-Lm, the addition of HK-*L. monocytogenes* also limited protective immunity. In a similar fashion, the addition of the unrelated phagosome-confined non-pathogenic bacterium *Bacillus subtilis* also attenuated protective immunity (Figure 1F). Finally, the addition of LLO-Lm-OVA to an inoculum of wt-*L. monocytogenes* also impaired protective immunity (Figure 1G). Similar observations were made during experiments utilizing Balb/c mice (data not shown). Thus, as few as 9×10^5^ CFU of phagosome-confined LLO-Lm-OVA added to an inoculum of cytosolic ActA-Lm-OVA during primary infection leads to a greater than 1000-fold increase in CFU following wild-type challenge.

### Phagosome-confined *L. monocytogenes* negatively impact the magnitude of the primary CD4 and CD8 T cell response

**Figure 2 ppat-1000568-g002:**
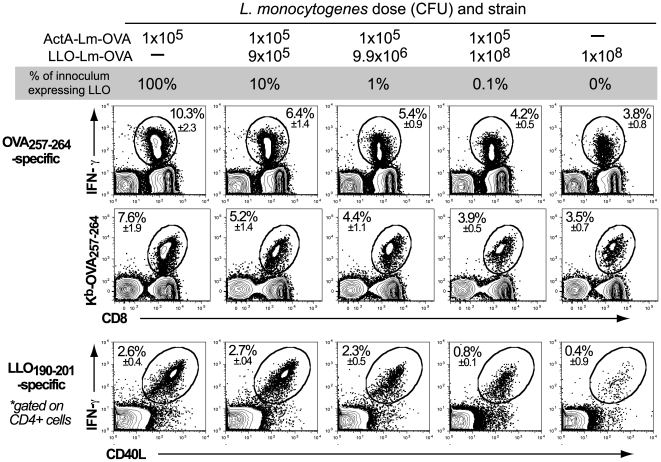
*L. monocytogenes* within a phagosome impairs the primary T cell response. Mice were infected with 1×10^5^ CFU ActA-Lm–OVA alone, or in combination with increasing doses of LLO-Lm-OVA. 7 days later, the frequency of OVA_257–264_-specific CD8 T cells and LLO_190–201_-specific CD4 T cells was determined by pentamer and IFN-γ intracellular cytokine staining. Total splenocyte number and absolute CD8 T cells per spleen were consistent between all groups. Values in each plot represent the mean±SEM of antigen-specific cells within the CD4 or CD8 population from 5 animals per group.

**Figure 3 ppat-1000568-g003:**
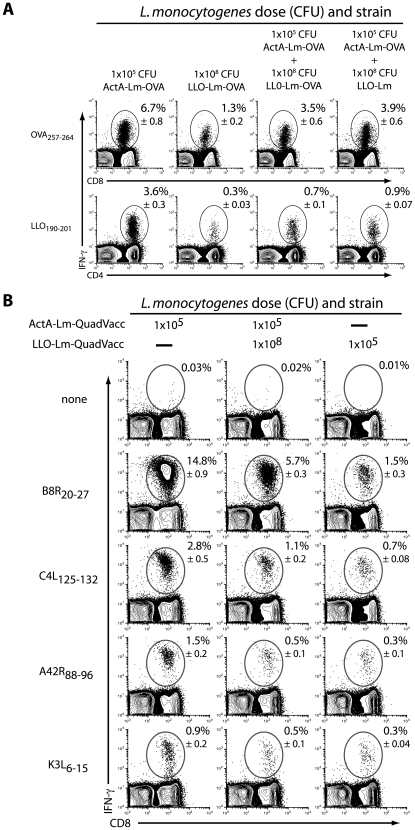
Suppression of the primary T cell response is antigen-independent. (A) Mice were infected with the indicated combinations of 1×10^5^ CFU ActA-Lm, 1×10^5^ CFU ActA-Lm–OVA, 1×10^8^ CFU LLO-Lm, and 1×10^8^ CFU LLO-Lm-OVA. 7 days later, the frequency of OVA_257–264_ -specific CD8 and LLO_190–201_ -specific CD4 T cells was determined by IFN-γ intracellular cytokine staining. (B) ActA-Lm and LLO-Lm *L. monocytogenes* were engineered to express 4 defined epitopes from vaccinia virus. Mice were infected intravenously with the indicated combinations of ActA-Lm-QuadVacc and LLO-Lm-QuadVacc. 7 days later, spleens were harvested and the frequency of CD8 T cells specific for each epitope was determined by IFN-γ intracellular cytokine staining. Total splenocyte number and absolute CD8 T cells per spleen were consistent between all groups. Values in each plot represent the mean±SEM of IFN-γ+ cells within the CD4 or CD8 population from 5 animals per group.

Given the role of CD8 T cells in protective immunity, we questioned how the addition of increasing numbers phagosome-confined LLO-Lm-OVA would impact the primary T cell response to a constant dose of ActA-Lm-OVA. Similar to our observations following wild-type *L. monocytogenes* challenge, the 10 to 1000-fold increase in the number of OVA-expressing bacteria did not improve the primary T cell response. Instead, the magnitude of the primary CD8 OVA_257–264_ and CD4 LLO_190–201_–specific response declined as the ratio of phagosome-confined to cytosolic bacteria increased (Figure 2). The frequency of OVA_257–264_-specific CD8+ T cells determined by IFN-γ staining was confirmed using K^b^-OVA_257–264_ multimers to rule out the existence of OVA_257–264_ -specific CD8+ T cells incapable of producing IFN-γ. To understand if suppression of the T cell response was antigen specific, we performed similar studies using LLO-Lm that did not express OVA (Figure 3A). These studies demonstrated that suppression was antigen-independent, as LLO-Lm expresses neither the OVA_257–264_ nor the LLO_190–201_ epitopes. Furthermore, the reduced magnitude of the primary response was independent of the class I-restricting allele or the affinity of the MHC-peptide interaction as observed using *L. monocytogenes* strains expressing four defined vaccinia virus-derived epitopes (Figure 3B) [36]. Therefore, the presence of LLO-Lm within a phagosome negatively impacts both the primary CD4 and CD8 T cell response to cytosolic ActA-Lm as well as protective immunity.

### Phagosome-confined *L. monocytogenes* limit inflammatory cytokine production

**Figure 4:**
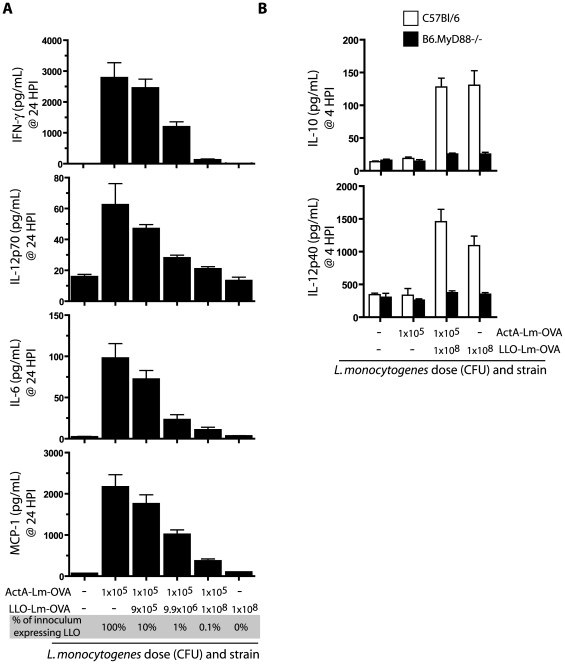
*L. monocytogenes* within a phagosome suppress the host inflammatory response to bacteria within the cytosol. (A) Mice were infected with 1×10^5^ CFU ActA-Lm–OVA alone, or in combination with increasing doses of LLO-Lm-OVA. Serum was collected 24 hours later and assayed for IFN-γ, IL-12p70, IL-6, and MCP-1. (B) C57Bl/6 and B6.MyD88−/− mice were infected with 1×10^5^ CFU ActA-Lm–OVA, 1×10^8^ CFU LLO-Lm-OVA, or the combination of both strains. Serum was collected 4 hours later and assayed for IL-10 and IL-12p40. Bars represent the mean and standard error of 5 mice per group.

The innate immune response during infection plays a critical role in shaping the ensuing adaptive response. Based on the observed suppression of CD4 and CD8 T cell responses, we hypothesized that the presence of phagosome-confined *L. monocytogenes* altered the inflammatory cytokine response to the cytosolic ActA-Lm strain. We compared serum cytokines between mice infected with ActA-Lm-OVA alone versus in combination with increasing numbers of LLO-Lm-OVA. Infection with combinations of LLO-Lm-OVA and ActA-Lm-OVA led to the dose-dependent reduction of serum IFN-γ, IL-12p70, IL-6 and MCP-1 relative to ActA-Lm-OVA alone (Figure 4A). Thus, LLO-Lm-OVA bacteria within a phagosome exert a negative effect on the pro-inflammatory response elicited by ActA-Lm-OVA within the cytosol. Because many vacuolar pathogens elicit a Th2-type cytokine profile [37], we questioned the ability of LLO-Lm-OVA to elicit cytokines that might limit the potency of the adaptive T cell response. Four hours post infection, serum IL-10 was detectable in mice immunized with LLO-Lm-OVA, either alone or in combination with ActA-Lm-OVA, and required the adapter protein MyD88 (Figure 4B). In addition, we detected high levels of IL-12p40 in the absence of heterodimeric IL-12p70, suggesting high levels of IL-12p40 homodimer were present in the serum (although we cannot rule out that p40 was complexed with p19 as functional IL-23). Thus, the addition of phagosome-confined LLO-Lm-OVA to an inoculum of ActA-Lm-OVA inhibits inflammatory cytokine production and corresponds with elevated levels of IL-10.

### Suppression of protective immunity by phagosome-confined *L. monocytogenes* is dependent on MyD88 and IL-10R signalling

**Figure 5:**
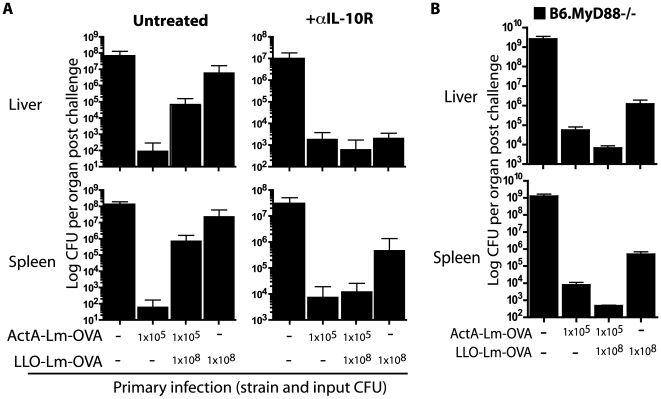
IL-10 receptor blockade during T cell priming prevents the suppressive effects of *L. monocytogenes* within a phagosome. Mice were infected with ActA-Lm–OVA, LLO-Lm-OVA, or the combination of both strains in combination with αIL-10R antibody. (A) 30 days post infection mice were challenged with a lethal dose of wt *L. monocytogenes*-OVA. Spleens were harvested 3 days later and CFU per spleen determined. Each bar represents the mean and standard error of 5 mice per group. (B) B6.MyD88−/− mice were infected with 1×10^5^ CFU ActA-Lm-OVA, 1×10^8^ CFU LLO-Lm-OVA, or the combination of both strains. 16 weeks later, mice were challenged with 1×10^3^ CFU wt *L. monocytogenes*-OVA. Spleens and livers were harvested 3 days later and CFU per organ determined. Each bar represents the mean and standard error of 3–5 mice per group. Data are from one representative experiment of two.

To examine the role of IL-10 in limiting the potency of the adaptive response to LLO-Lm-OVA, mice were infected with ActA-Lm-OVA and LLO-Lm-OVA in combination with an antagonist IL-10 receptor antibody (anti-IL-10R) [38]. This regimen permits blockade of IL-10 signalling during priming while maintaining an intact immune system during challenge. Only the highest dose of LLO-Lm-OVA was used in combination with ActA-Lm-OVA, a combination that led to the greatest suppression of inflammatory cytokines and protective immunity (Figures 1–4). On day 30, mice were challenged with wild-type-*L. monocytogenes*-OVA and protective immunity assessed 3 days later. Impressively, mice co-infected with LLO-Lm-OVA and ActA-Lm-OVA in the presence of IL-10R blockade demonstrated equivalent protection against wt-*L. monocytogenes*-OVA challenge as anti-IL-10R treated mice infected with ActA-Lm-OVA alone (Figure 5A).

IL-10 production following LLO-Lm infection was MyD88-dependent; therefore we questioned whether LLO-Lm would limit ActA-Lm-induced protective immunity in mice lacking MyD88. Similar to the results following IL-10R blockade, MyD88−/−mice immunized with the combination of ActA-Lm-OVA and LLO-Lm-OVA were protected against a lethal challenge with wt *L. monocytogenes* (Figure 5B). Thus, in the absence of MyD88 signalling, the ability of phagosome-confined *L. monocytogenes* to limit the adaptive response to *L. monocytogenes* within the cytosol is eliminated. Together, these results demonstrate that the innate immune system discriminates between pathogens that reside in distinct subcellular locations, and via a MyD88- and IL-10-dependent mechanism, impacts the potency of the adaptive immune response.

## Discussion

How the immune system differentiates between pathogenic and non-pathogenic microbes and translates this information into an appropriate immune response is not completely understood. Previous reports have shown that intracellular bacteria, including *L. monocytogenes*, activate a unique host cytosolic surveillance pathway of innate immunity while extracellular bacteria do not [9],[16],[39],[40]. These studies led to our original hypothesis that activation of the host cytosolic surveillance pathway provided an explanation to the observations that heat-killed and LLO- *L. monocytogenes* fail to immunize mice to subsequent challenge [8],[41]. Our original goal in these studies was to improve the potency of CD8 T cells responding to phagosome-confined LLO-Lm-OVA by complementing it with a cytosolic *L. monocytogenes* strain. Surprisingly, we found that the presence of phagosome-confined LLO-Lm negated the innate response to the cytosolic *L. monocytogenes* strain and ultimately compromised long-lived protective immunity (Figure 1). These results suggest that although recognition of microbial constituents within the cytosol may elicit cytokines that improve the cellular immune response, it is the exit from the phagosome that permits this inflammatory response to take place.

Our data indicate that the potency of the adaptive T cell response is incrementally altered as the ratio of intracellular to phagosome-confined bacteria changes. Both ActA-Lm and LLO-Lm are similarly distributed amongst phagocytic cells in vivo [42]. Furthermore, because ActA-Lm cannot polymerize host-cell actin, neither strain will spread into neighbouring cells [43]. Thus the ratio of ActA-Lm to LLO-Lm will not alter the cell types that interact with the bacteria. To avoid overwhelming the innate immune system with *L. monocytogenes*, we decreased the dose of ActA-Lm to 1×10^5^ CFU (from the standard 0.1×LD_50_ dose of 1×10^7^ CFU) then added 10–1000-fold of LLO-Lm to the inoculum. Using these doses, the input CFU exceeded 1×10^7^ CFU in only the highest dose group (1×10^5^ CFU ActA-Lm+1×10^8^ CFU LLO-Lm). Therefore, the loss of protective immunity observed following infection with 1×10^6^ and 1×10^7^ total CFU could not be explained by the increase in total CFU alone, as 1×10^7^ CFU of ActA-Lm has been shown many times to elicit complete protective immunity to wild-type challenge [34],[44].

The innate response to infection plays a pivotal role in shaping the adaptive immune response [29]. Suppression of cytokines and chemokines following recognition of microbial PRRs could impact the adaptive response via multiple mechanisms [45]. Chemokines produced at the site of infection facilitate the infiltration of neutrophils, macrophages and dendritic cells to the affected tissues [3],[25]. Neutrophils and activated macrophages are critical for controlling bacterial replication, while dendritic cells are required for processing and presentation of microbial peptides [46]. Dendritic cells presenting bacterial antigens undergo maturation in response to proinflammatory cytokines. This maturation step is critical for modifying many aspects of dendritic cell function, including: the expression of specific proteasome subunits and thus, the repertoire of peptides available for presentation [47],[48]; the density of MHC and co-stimulatory molecules on the cell surface [49]; and secretion of chemoattractants which recruit naïve T cells into the secondary lymphatics. In addition, inflammatory cytokines can act via direct co-stimulation of T cells during priming [50]. Thus, by virtue of its inability to escape from the phagosome, LLO-Lm alters the innate inflammatory landscape and ultimately, the potency of the *Listeria*-specific T cell response.

The impact of cytokines on T cell potency is complex, as the effect of a specific cytokine can be dependent on location, context, and concentration. In previous studies, IL-10 was necessary for optimal T cell memory following *L. monocytogenes* infection [28]. However, elimination of IL-10 signalling from only CD8 T cells improved the magnitude and function of the response [26]. In agreement, when mice were immunized with ActA-Lm during IL-10R blockade, a small but reproducible increase in liver and spleen cfu was observed after wild-type challenge (Figure 5A). Conversely, when the IL-10 receptor was blocked during immunization with LLO-Lm-OVA, protective immunity improved, resulting in 2–3 logs fewer CFU following lethal challenge. While IL-10 was detectable in low, but reproducible amounts following immunization with LLO-Lm-OVA, we were unable to measure IL-10 following immunization with ActA-Lm (Figure 3B). Furthermore, serum IL-10 was only consistently detectable using the highest dose of LLO-Lm, 1×10^8^ CFU. Measuring serum IL-10 in the presence of IL-10R blockade prevents IL-10 uptake and greatly improves the sensitivity of this assay. This approach increased serum IL-10 following LLO-Lm-OVA immunization 5–10-fold, while IL-10 in ActA-Lm immunized mice remained undetectable (data not shown). Thus, while IL-10 certainly impacts T cell potency following infection with wild-type- or ActA-Lm, its concentration is far below that measured after immunization with LLO-Lm-OVA. When assessing the role of IL-10R signalling on the suppression of memory T cell function, we chose to use the highest dose of LLO-Lm (1×10^8^ CFU) in combination with 1×10^5^ CFU ActA-Lm. This dose combination, which provided the most consistent levels of serum IL-10, also provided the greatest amount of suppression (Fig 1–3). Thus, while we demonstrated suppression of the T cell response by a 1000-fold range of LLO-Lm, the highest and most inhibitory dose was chosen to assess the dependence on IL-10R signalling. The biological activity of these low concentrations of IL-10 suggest that its effects are locally restricted, requiring only minute concentrations but within a defined location. One possibility is that as the concentration of systemic IL-10 increases, its impact on the T cell response changes from positive to negative regulator. This functional switch might be attributed to differences in sensitivity to IL-10, or other cytokines produced within the same microenvironment. These studies suggest that during *L. monocytogenes* infection, IL-10 acts as a negative regulator of T cell potency in CD8 T cells, while acting as a positive regulator of cellular immunity via its effects on other cell types.

The results from these studies are significant both to the fields of microbial pathogenesis and vaccinology. Understanding how microbes interact with the innate and adaptive immune system is critical for controlling their pathogenic effects. Vaccines remain one of the most cost-effective tools for preventing disease and improving health worldwide. While vaccines that elicit humoral immunity have been relatively straightforward to develop, vaccines intended to elicit robust cellular immunity, such as those needed to combat HIV and tuberculosis, have remained elusive [51]. These difficulties may be in part due to our poor understanding of how the adaptive immune response is regulated [52]. Modern approaches to developing these vaccines have used killed or attenuated forms of otherwise pathogenic organisms in hopes of eliciting the appropriate immune response without overt disease [53]. Upon observing an inadequate immune response, a common next step is to add an adjuvant to the vaccine to improve its immunogenicity, or to explain its impotence as a lack of positive inflammatory signals [54]. Our studies show that recognition of microbial products within defined cellular compartments can negate inflammation and limit the potency of the cellular immune response even when numerous proinflammatory signals are present. This result may explain why the addition of adjuvants to safe but ineffective vaccines intended to elicit cellular immunity is often unsuccessful. Furthermore, these studies add to the emerging field of microbial subversion of innate and cellular immunity and serve as a primer for defining new regulatory signalling pathways [55].

These studies shed new light on the classic observation that only microbes entering the host cell cytosol lead to a productive antigen-specific CD8 T cell response. It is not simply a case of inefficient antigen processing and presentation or the inability to activate the cytosolic surveillance pathway; the innate immune response to bacteria residing within a phagosome negates the innate and adaptive response to otherwise stimulatory bacterial products. Additional experiments are required to define the exact receptor-ligand interactions that take place within a phagosome, as well as to identify other cytokines and chemokines that may impact inflammation and T cell potency in this scenario. Understanding these negative regulatory pathways will be pivotal for the rational design of safe and potent vaccines that elicit long-lived T cell-mediated immunity.

## Methods

### Ethics statement

All animal protocols were approved by the Earle A. Chiles Research Institute, University of California, Berkeley or the Anza Institutional Animal Care and Use Committee.

### Mice, bacterial strains and infections

6–10 week old C57Bl/6 mice were purchased from Charles River Laboratories (Wilmington, MA). B6.MyD88−/− mice were bred at our facilities. *L. monocytogenes* strains ActA-Lm-OVA and LLO-Lm-OVA were constructed as previously described [8]. Both strains secrete full-length chicken ovalbumin fused to the first 441 amino acids of LLO and controlled by the *hly* promoter. ActA-Lm-QuadVacc and LLO-Lm-QuadVacc were constructed using an ActAN100 fusion with the vaccinia virus derived epitopes B8R_20–27_ TSYKFESV K^b^-restricted; C4L_125–132_ LNFRFENV K^b^-restricted; A42R_88–96_ YAPVSPIVI D^b^-restricted; K3L_6–15_ YSLPNAGDVI D^b^-restricted [36],[56]. Bacteria were grown to midlog in brain-heart infusion broth, washed in PBS, then injected intravenously in 200 µL total volume. Mice were injected intravenously with 250 µg anti-IL-10R (CD210, clone 1B1.3a, BD Bioscience, San Diego, CA) 2 hours before *L. monocytogenes* infection.

### Lethal challenge and bacterial enumeration

Mice infected 30 or 60 days prior were challenged with 2×LD_50_ (1×10^5^ CFU) wild-type *L. monocytogenes*-OVA (L4056-OVA). 3 days later, spleens and livers were homogenized and serial dilutions plated on BHI-strep agar plates for enumeration. Experiments using ActA-Lm-Erm^R^ were plated in duplicate using BHI-strep and BHI-strep-erm agar. In vitro growth was determined in bone-marrow derived macrophages adhered to coverslips at the indicated timepoints. Coverslips were vortexed in lysis buffer and plated on strep-erm agar to enumerate ActA-Lm-Erm^R^ bacteria.

### Flow cytometry

Spleens were harvested, dissociated, and red blood cells removed by ammonium chloride lysis buffer (Sigma, St. Louis, MO). Following 5 hours of restimulation with the relevant peptide in the presence of brefeldin A, cells were stained with anti-CD4 (clone GK1.5 , eBioscience, San Diego, CA) and anti-CD8 (clone 53-6.7, BD Biosciences), then fixed, permeabilized and stained for intracellular IFN-γ. (clone XMG1.2, eBioscience) [8]. Pentamer staining was performed using K^b^-OVA_257–264_ pentamers conjugated to APC (ProImmune Ltd, Bradenton, FL). Data was acquired on a FACSCanto flow cytometer (BD Bioscience) and analyzed using FlowJo software (Treestar, Ashland, OR).

### Serum cytokines

Serum was analyzed using the CBA Mouse Inflammation Kit (BD Biosciences) and FACSCanto flow cytometer (IL-10, IFN-γ, MCP-1, IL-6, IL-12p70), and the LincoPlex Multiplex Assay (Millipore, Billerica, MA) and Luminex 100 instrument (IL-12p40). Time points of 4 and 24 hours post infection were chosen as the peaks of the early and late cytokine response, determined during a kinetic analysis of cytokine production [57].
